# The historical trajectory of a coffee agri-food system: A case study in Oaxaca, Mexico

**DOI:** 10.1007/s13280-023-01893-6

**Published:** 2023-07-25

**Authors:** Alejandra Ramírez-León, Veronique Sophie Avila-Foucat, Driss Ezzine-de-Blas

**Affiliations:** 1 Postgraduate of Sustainability Sciences, National Autonomous University of Mexico (UNAM), Oriente 163 Número 234, Col. Campestre Aragón, Alcaldía Gustavo A. Madero, C.P. 07530 Mexico City, México; 2 Economics Research Institute and National Coastal Resilience Laboratory, National Autonomous University of Mexico (UNAM), Circuito Mario de la Cueva, Ciudad de la Investigación en Humanidades, Ciudad Universitaria, C.P. 04510 Mexico City, México; 3 Centre de Coopération Internationale en Recherche Agronomique Pour le Développement (CIRAD), Montpellier, France

**Keywords:** Adaptive cycle, Adaptive strategies, Agri-food systems, Coffee, Historical trajectory, Social–ecological systems

## Abstract

Agri-food social-ecological systems (AFSES) embrace complex interactions and processes of food production, processing, and commercialization that are subject to constant changes. This study develops a heuristic approach using the adaptive cycle (AC) and a transformation potential measure to identify the historical trajectory of a coffee AFSES at a watershed scale in Copalita, Mexico, over 40 years from 1980 to 2020. Primary information was collected through semistructured interviews. The results show that the system interactions depend on economic, social, and environmental stressors and shocks affecting different temporal and spatial scales. The cumulative effects of driving forces and adaptive strategies have influenced the system to not complete the AC phases. Additionally, the results show that some adaptive strategies can become new stressors with time. Driving forces, adaptive strategies, tipping points, trade-offs and interactions within the AFSES could be identified as the main aspects defining system resilience.

**Supplementary Information:**

The online version contains supplementary material available at 10.1007/s13280-023-01893-6.

## Introduction

Agri-food systems are the result of complex interactions between society and nature, shaping a social-ecological system (SES) that provides many ecosystem services to satisfy human needs and household livelihoods (Valbuena et al. 2013). Agri-food social-ecological systems (AFSES) include food production, transformation, distribution, and consumption processes in which interactions take place defining the SES trajectory in time and space. Biophysical conditions for production and markets have been identified as some of the main forces of change, but AFSES also respond to changing contexts, such as human and technological resources, input costs, public policies, and consumer preferences (Campanhola and Pandey, 2019), as well as external driving forces such as climate change and social conflicts (Huber-Sannwald et al. 2012). In addition, the effects of the system interactions and driving forces might be different in time and space, and especially when linking production to water resources and climate, the watershed scale becomes relevant to understanding how spatial heterogeneity generated by local biophysical factors influences decisions that transform the trajectory of the SES (Enfors 2013; Mokondoko et al. 2022).

The AFSES are typically influenced by multiple stressors and shocks (agro-ecological, economic and political-social driving forces) causing changes at various levels (farm, watershed, region, country) (Darnhofer 2014). Consequently, the AFSES adjusts its responses to external and internal driving forces to evolve and learn to develop toward a pathway or trajectory (Preiser et al. 2018) that can be within a stability domain or moving from one state to another. These pathways are also the result of the historical interactions between components of the system (environmental-ecological, economical-technical, and political-social components) and the accumulated effects of shocks and stressors (Duru and Therond 2015). The system is in a transition when crossing between two states and is in a transformation when it has crossed a threshold, implying that the system has lost its resilience (Folke et al. 2010). For example, when diseases and pests appear, the farmer could combat them to continue cultivating, processing, and marketing his crop. On the other hand, if the magnitude of the damage exceeds the farmer's capabilities, he leaves that crop to carry out new activities that will change the ecological and environmental conditions and his livelihood. Resilience theory explains that if a system maintains the same state, it means that the system preserves its main structure, function and identity, which resides in the continued presence, in both space and time, of key components and relationships without crossing a threshold (Cumming and Collier 2005). Thus, studying SES resilience implies understanding how a system evolves without losing the essential features that characterize it.

**Fig. 1:**
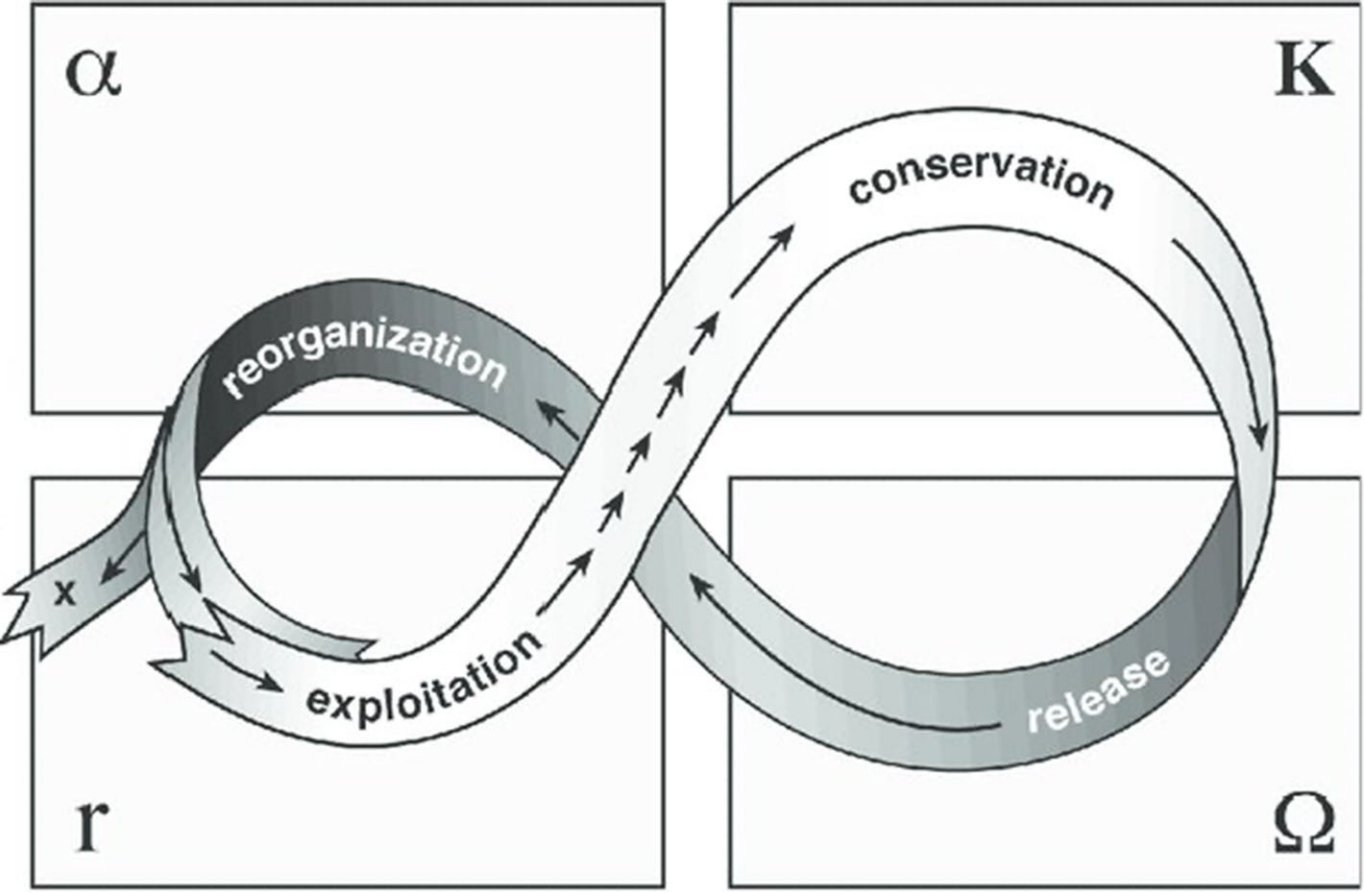
Adaptive cycle. Source: Gunderson and Holling 2022

The adaptive cycle (AC) has been used as a heuristic theory of change to study the trajectories and resilience of SES (Folke 2016). The AC (Fig. 1) describes the endogenous dynamics of SES generated by internal processes of self-organization and evolution over time through the succession of four phases: growth, conservation, collapse, and renewal (Holling 2001). During the growth or exploitation phase (r), the system enters a slow and cumulative progressive cycle. In the conservation phase (K), resources become increasingly blocked, and the system becomes progressively less flexible and receptive to external disturbances. The loop formed by r-K consists of a self-regulating system that makes a system responsive and capable of adapting to both internal and external changes; it is marked by continuous accumulation of different forms of capital facilitated by self-reinforcing feedback loops between the system's components, which leads to accumulating resources, know-how and welfare (Kuhmonen and Kuhmonen 2013, p. 3). These conditions maintain the system with a certain range of variability or within a certain domain of attraction, that is, it maintains the same characteristics that identify it (Cabel and Oelofse 2012). This phase is followed by a chaotic collapse and a release phase (Ω), which quickly gives way to a reorganization or renewal phase (α), in which innovation and new opportunities are possible and consequently allow the system to stay in the same state and be resilient. The reorganization phase is the degree to which farmers, consumers, and other stakeholders can organize themselves; any configuration that they create is more likely to contribute to the overall system resilience in the long term because it was created by their initiative in response to a real need (Holling 2001; Cabel and Oelofse 2012).

This evolutionary process depends on the intensities of shock and stressors and on the conditions and capacities of the system to respond, absorb disturbances and reorganize to maintain the same structure and identity (Walker et al. 2004; Folke et al. 2010). Three types of capacities have been recognized: persistence or absorptive capacity (ability to resist effectively, absorbing the shock impacts without changing their function, status, or state); adaptability or adaptive capacity (incremental changes and adaptations that people undergo to continue functioning in response to a shock or growing stress), and transformability or transformative capacity (human actions taken to create or enable a fundamentally new system) (Béné et al. 2016; Sinclair et al. 2017).

These three capacities depend on the initial conditions of the systems, such as assets or ecosystem services but also on the agency of actors (executed by people who make decisions) influencing the system through different types of responses linked to different intensities of shock or change (Béné et al. 2016). For example, persistence emerges from coping strategies by which its members moderate or buffer the impacts of shocks on their livelihoods and basic needs. Adaptive capacity refers to incremental changes without major changes to the way SES operates (adaptive strategies). The transformative capacity emerges when the changes required in response to shocks or stresses are so large that the system is altered, changing its functioning or structure (Béné et al. 2016).

The AC has been useful to identify drivers of change, key variables, and the effects of stressors on SES in studies about forest management (Beier et al. 2009), agropastoral subsystems (Rasmussen and Reenberg 2012), and ecosystem services (Pérez-Orellana et al. 2020). Other studies related to farming SES, such as Abel et al. (2006), Beier et al. (2009), and Antoni et al. (2019), have used this approach to analyze the evolution of SES and changes of state, as well as their components and relationships. In addition, other studies have used AC to address the resilience of AFSES (Cabel and Oelofse 2012; Santos Prado et al. 2015; Sinclair et al. 2017; Darnhofer et al. 2010; Meuwissen et al. 2019). Darnhofer et al. (2010), Cabel and Oelofse (2012) and Meuwissen et al. (2019) proposed frameworks to assess resilience as well as indicators related to the attributes of a resilient AFSES, such as diversity, modularity, reflective and shared learning, social self-organization, and ecological self-regulation, among others.

All the cited studies that used AC to address the resilience of AFSES agree on the importance of analyzing the different types of changes and their effects, as well as the different intensities in time and space. They highlight that those interactions at different spatiotemporal scales can generate unpredictable effects that will inevitably modify the future trajectory of a system; however, few studies specify spatial effects within a watershed. They also point out the relevance of identifying the thresholds and/or tipping points and the role played by the agency and governance to achieve desirable system transitions. Santos Prado et al. (2015) concluded that the analysis of trade-offs among components needs to be considered, balancing both the positive and negative aspects and considering the cross-scale connections, and highlighted the need for a more quantitative method to determine transitions and thresholds. Within the revised literature using the AC approach, historical data and qualitative interpretation have been used to identify transition phases before a complete transformation.

The AC has demonstrated its usefulness in explaining how SES experience periods of gradual change interrupted by shorter episodic disturbances that may reconfigure the system (Darnhofer et al. 2010; Gunderson et al. 2022). In addition, it allows us to identify relationships and feedback between natural and social variables. This makes it possible to identify how internal and external driving forces affect the system and its trajectory over time, which influences and shapes current and future trends (Preiser et al. 2018). The analysis of the historical trajectory of SES can provide important information to understand the actual conditions and the challenges imposed for resilience and sustainability in planning and decision-making (Nguyen et al. 2019). However, few studies using AC have deepened the relationship between shock and stressor effects on the system, causing adaptive capacities to address system resilience. Thus, we used AC as a theoretical point of reference to identify transition and transformation and the role played by adaptive capacity related to them. The analytical-theoretical framework proposed combines the AC and the concepts of dimensions of change (Fazey et al. 2018) to characterize quantitatively the effects of driving forces to determine when transitions happen.

The case of the coffee AFSES is used as empirical evidence due to its economic importance and its wide geographical distribution. Coffee is commercially grown in tropical developing countries, mainly in conditions of marginalization and poverty (Bacon 2005; Olsson et al. 2014) where climate and biophysical factors are key aspects; its international trade is dominated by a few transnational companies with profits concentrated in processing and commercialization, which are estimated to surpass USD 200 billion (ICO 2019). This in turn is proof of the complexity of this AFSES, and learning about its trajectory can generate many insights into resilience theory.

**Fig. 2:**
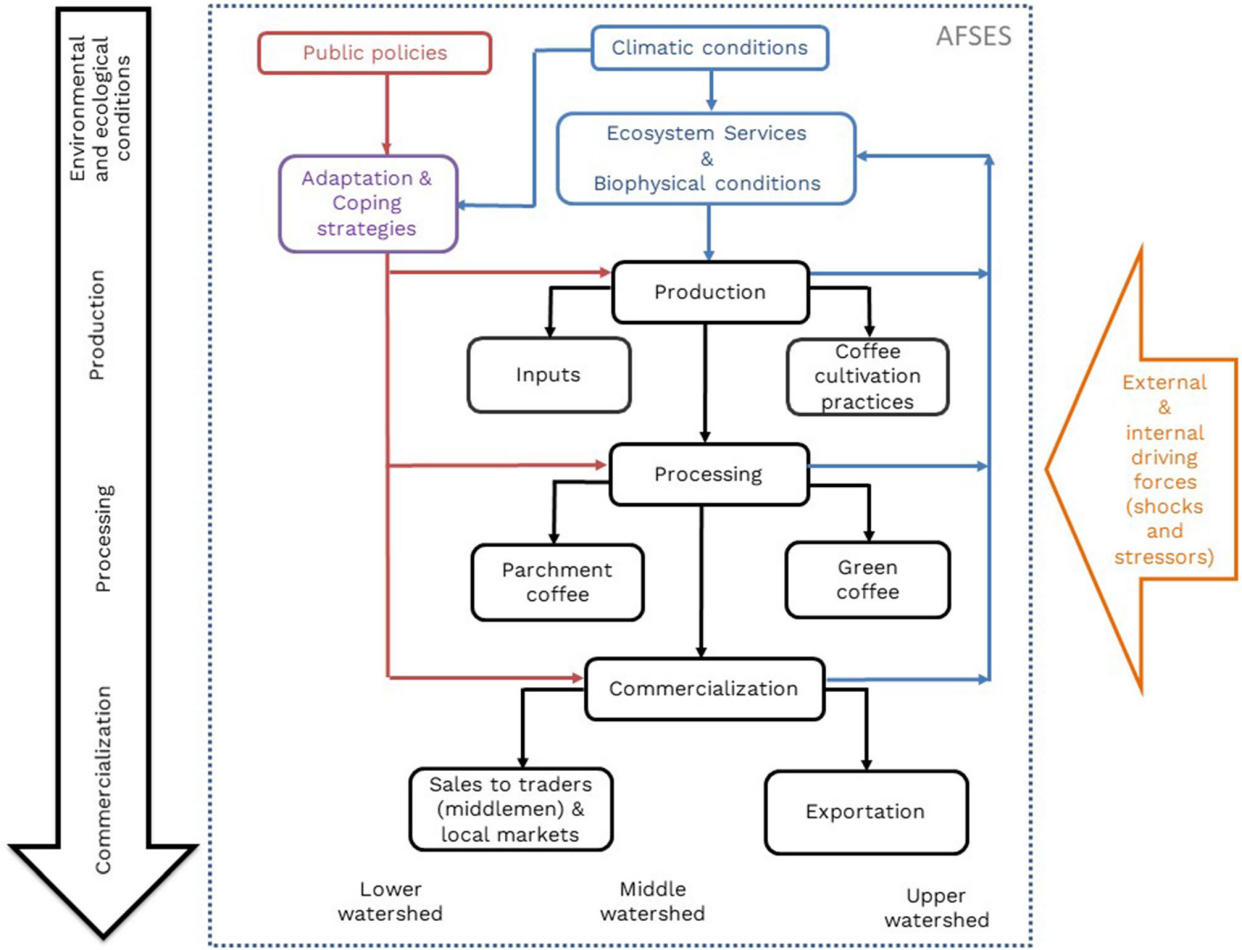
Coffee agri-food social-ecological system (AFSES) of Copalita Huatulco Watersheds (CHW). Source: Own elaboration

Specifically, the case study is located in the Copalita-Huatulco watersheds (CHW) in Mexico, where coffee production represents 24% of Oaxaca state production, being the fourth largest coffee region in Mexico (SIAP 2021). Here, shade coffee production predominates as an agroforestry system that contributes to regulation ecosystem services such as regulation of local climate, conservation of soil fertility, and biological regulation of water flows, among others (FAO 2020). The global conditions related to the coffee market are characterized by instability of markets, affectations by climate change, and limited bargaining power of farmers in the coffee value chain, have influenced the local conditions distinguished by price speculation, an increase of temperature, and poverty among coffee smallholders. These conditions suggest that CHW has suffered from tipping points that have taken the system from a condition of relative stability to a context of crisis and uncertainty that we want to determine. Figure 2 represents the coffee AFSES located in the CHW; it shows the four components of the system and the interactions among them, as well as its geographic location in the watershed.

This study aims to analyze the historical trajectory of the coffee AFSES of the CHW from 1980 to 2020 to understand the system dynamics and possibilities for enhancing its resilience. We ask the following questions: Does the coffee AFSES trajectory follow the AC? What are the tipping points defining the AC of a coffee AFSES, and does the method proposed help to identify them? What are the main adaptive capacities identified? The coffee AFSES could have recovered by maintaining the same structure and returning to the coffee bonanza, or it could have transformed into a system with a completely new structure and characteristics where coffee would have been abandoned and new economic activities carried out to have a more stable state. This paper aims to contribute to the literature assessing trajectories and resilience in AFSES at a watershed scale by identifying the transition phases and tipping points using the AC and a transformability potential. This knowledge will improve our understanding of the management of complex AFSESs and can also be used in other similar systems around the world and provide information for local adaptation planning. In the 1980s, major international coffee crisis occurred, which influenced a process of socioeconomic restructuring in the CHW that was exacerbated by environmental events such as hurricanes (Jaffee 2019). We choose the start of this disruptive event that triggered a series of abrupt changes (Speelman et al. 2014; Nayak and Armitage 2018; Nguyen et al. 2019) to establish the study period from 1980 to 2020.

## Method

### Description of the area of study

**Fig. 3:**
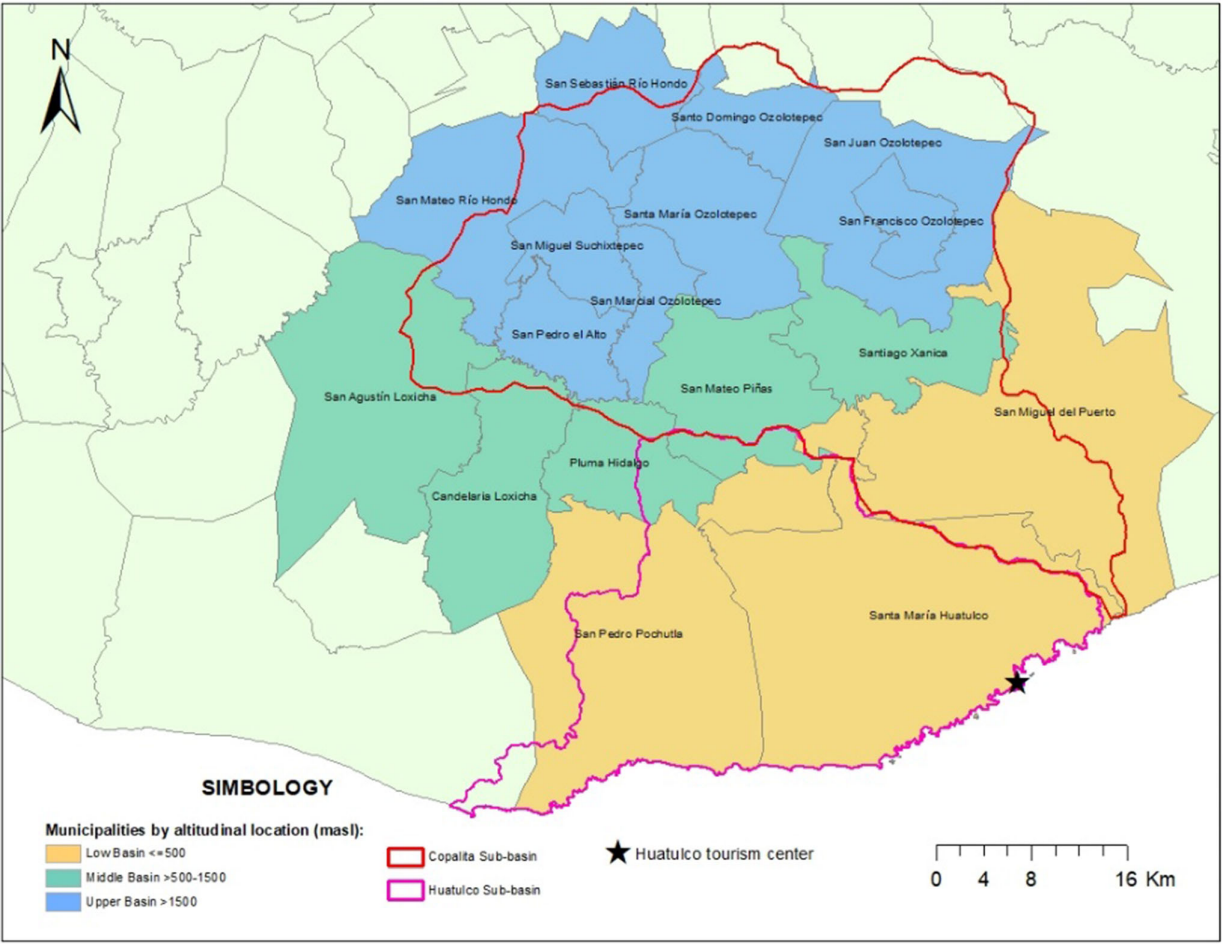
Copalita-Huatulco Watersheds (CHW). Source: Own elaboration

The Copalita-Huatulco watershed (CHW) is located on the Pacific coast of Mexico in Oaxaca state, it covers approximately 187,576 hectares and is formed by the Copalita and Huatulco subwatersheds (Fig. 3). Based on the type of vegetation, we established three areas of the watershed: the upper part ranged between 1,501 and 2,900 m, the middle part ranged between 501 and 1,500 m, and the lower part ranged between 0 and 500 m. In the upper part, pine-oak forests prevail; in the middle part, there are sub evergreen and mesophyll forests; and in the lower part, there is deciduous forest (SAGARPA and SEDAPA 2015). Since the ecosystems vary according to the altitudinal range, there are economic activities that can be better developed in specific parts and not in the entire watershed. The CHW is the home of nineteen municipalities with 181,715 inhabitants (INEGI 2020). In 2015, 87% of the population of the CHW suffered from poverty and had income below the welfare line (CONEVAL 2020).

**Fig. 4:**
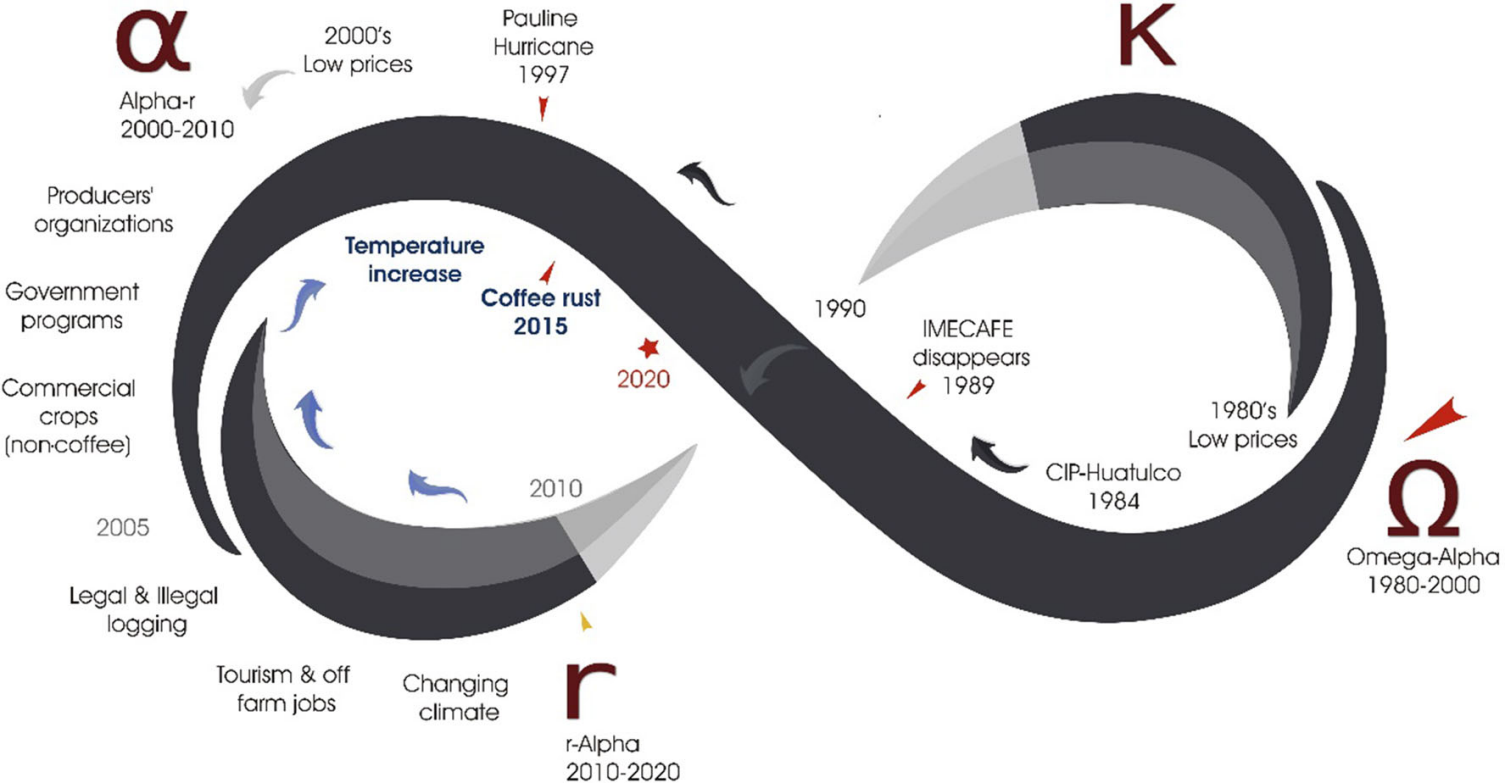
Trajectory of the Copalita-Huatulco SES based on the AC: 1980–2020. Source: Own elaboration. The large red arrow marks the beginning of the cycle in the 1980s, and the red star marks its end in 2020. The red arrows indicate the shocks that have affected the AFSES. The low prices of coffee have stayed constant over the 40-year period. In the 2000s, stressors accumulated because of the effects generated previously. In 2010, the cycle continued toward a phase of recovery that was interrupted and caused it to reverse; this setback is indicated by the yellow arrow

The watershed provides favorable conditions for coffee cultivation (INEGI 1997), which was an economic mainstay in the region from the nineteenth century until the second half of the twentieth century. In the watershed, there are different growing conditions for coffee. The upper basin is home to 30% of the cultivated coffee area, and forestry is also an important economic activity there. The middle basin presents 50% of the cultivation area for coffee dominated by landowners with more than ten hectares (50% of coffee farmers). In the lower basin is 20% of the cultivated coffee area. Small producers are located throughout the basin and they are owners of 37% of the cultivated area, comprised of 8,240 households that own up to five hectares of land. Only 12% of producers are medium farmers with plots between 5 and 10 hectares (SAGARPA and SEDAPA 2015). Fishing and livestock activities are in the middle and lower parts, and self-subsistence crops are cultivated throughout the watershed. Tourism and off-farm jobs are more important within the coastal limits of the basin due to the Huatulco resort.

### Data collection

We conducted semistructured interviews with stakeholders using the snowballing method (Corbin and Strauss 2012). The first contact was with a representative of a nongovernmental organization that provides advice on agroforestry systems; he recommended speaking with a retired researcher who specialized in the cultural and political changes in the coffee sector in the state of Oaxaca. She helped us to identify the first key actors who could provide historical information about the CHW. The face-to-face visits to conduct interviews began in July 2019 and were repeated in October, November, and December 2019. The last interviews were carried out by phone in March 2020 due to the beginning of the COVID-19 pandemic.

An interview applied face to face had an average duration of 90 min because several people were excited to narrate their experience in the coffee sector and the problems they faced. Phone interviews had an average duration of 50 min. The objective of the research was explained to each participant and at the time of the interview, authorization was requested to record the conversation under conditions of anonymity and confidentiality. We conducted thirty interviews that we transcribed for analysis. The respondents were leaders of producer organizations (n = 4); entrepreneurs who promote economic diversification (n = 3); representatives of nongovernmental organizations (NGOs) (n = 10); delegates of government (n = 8); researchers (n = 2); and coffee farmers (n = 3). The people interviewed have a position of leadership in political, social, environmental or economic issues related to coffee activity in the CHW; therefore, their views represent the systemic changes of the coffee AFSES.

When the interviewee was a farmer, the first section of questions focused on characterizing his or her community: the main economic activities, the main environmental problems, and their causes. In the case of institutional representatives, the first section asked about the functions of their organization and the type of support actions provided regarding coffee. In the second section, the main stressors and shocks were identified by asking what events affected coffee, when they happened, the causes and consequences and what people did to confront them. Secondary information was consulted to complement the information given by the interviewees. The complete questions are in Appendix S1, and examples of relevant testimonies are in Appendix S2.

### Data analysis

Qualitative content analysis was used to classify the collected information into more interpretable units of analysis (Corbin and Strauss 2012; Abela 2012). This classification was carried out using three steps to 1) identify stressors and shocks based on their effects on spatial distribution and time frame; 2) analyze the effects over the components of the AFSES (described in Fig. 1) in terms of spatial distribution and time frame; and 3) identify the adaptive strategies related to them. The coding system used to classify the information is in Appendix S3. This analysis was carried out using MAXQDA version 20.4.0.

The identification of stressors and shocks was based on their impacts in terms of the spatial and temporal effects within the AFSES: effects may entirely or partially (upper, middle or lower part) cover the watershed and may be felt over the short term (several months to a few years), long term (several years or decades), or ongoing (initiated in the past manifested in the present and with no identified conclusion). These data were classified using the categories: driving forces; components of the AFSES; and processes, situations and actors. A shock was defined by a short-term acute event with a rapid onset and a typically short duration, while stressors were usually chronic with a slow onset and a typically protracted duration (Sagara 2018). The effects were assessed using the concepts *of breadth* and *intensity*. Breadth is related to the spatial presence of the impacts on the watershed, and intensity is the temporality of the effects quantifying their time frame.

Through the temporal and spatial scale categories, we identified the data that allowed us to establish breadth and intensity for each stressor and shock; the results obtained were used to attain a matrix that quantified those concepts. In this matrix, the values of breadth were established through a binary measure of presence-absence (1-0); if a shock or stressor affected any component of the AFSES in any of the three parts of the watershed, we assigned a value of 1. If all parts of the watershed were affected, the maximum value obtained was 3. Intensity was weighted on a scale between 1 and 3, where the highest value (3) indicates a definitive and irreversible effect (when the effects are short-term and there is no way to reverse them), followed by incremental effect (2) (when the effects started at a point in the past, were maintained in the long term or are still manifesting), or temporal effect (1) (when the effects started and ended in a specific period). The sum of breadth and intensity represents the total effect of each stressor and shock on the AFSES, called the transformability potential (TP), which was adapted from the concept of transformability.

The TP supports the identification of the transition in the AC through a scale defined as very strong or irreversible with a TP between 20 and 24 points; strong with a TP between 15 and 19; middle with a TP between 10 and 14; and weak with a TP between 5 and 9. The highest range (very strong or irreversible) corresponded to an irreversible transformation in which the driving force triggered the beginning of a new AC in the AFSES (Ω); the next ranges (strong, middle, and weak) represented the push of the driving force to advance in the succession of the AC to the next phases (*α*, *r*, *K*) until reaching together a new phase of transformation (Ω). For example, Hurricane Paulina was identified as a shock and its breadth and intensity were calculated, whose values were 6 for both cases, giving a TP equal to 12. According to the defined scale, Hurricane Pauline had a medium TP (between 10 and 14), since it did not affect the entire basin or all the components of the system to the same extent. The TP of each driving force was used to attain a matrix to identify the AC of the system. According to the defined scale, Hurricane Paulina had a medium TP (between 10 and 14) since it did not affect the entire basin or all the components of the system to the same extent. The third step was to describe adaptive strategies through the choices and activities that individuals and groups made in response to stressors and shocks. The subcategory adaptive and coping strategies was used to identify those elements and obtain a matrix that summarizes the driving forces, their effects, and the adaptive strategies related to them.

## Results

The AFSES has transitioned through three phases of the AC: **from crisis to rearrangement** (Ω-α phase) between 1980 and 2000; **reconfiguration and innovation** (α-r phase) between 2000 and 2010; and **reorganization to new arrangements** (r-α phase) between 2010 and 2020. Table 1 summarizes the TP of shocks and stressors of different natures and how they influence the transition in the AC.

**Table 1:**
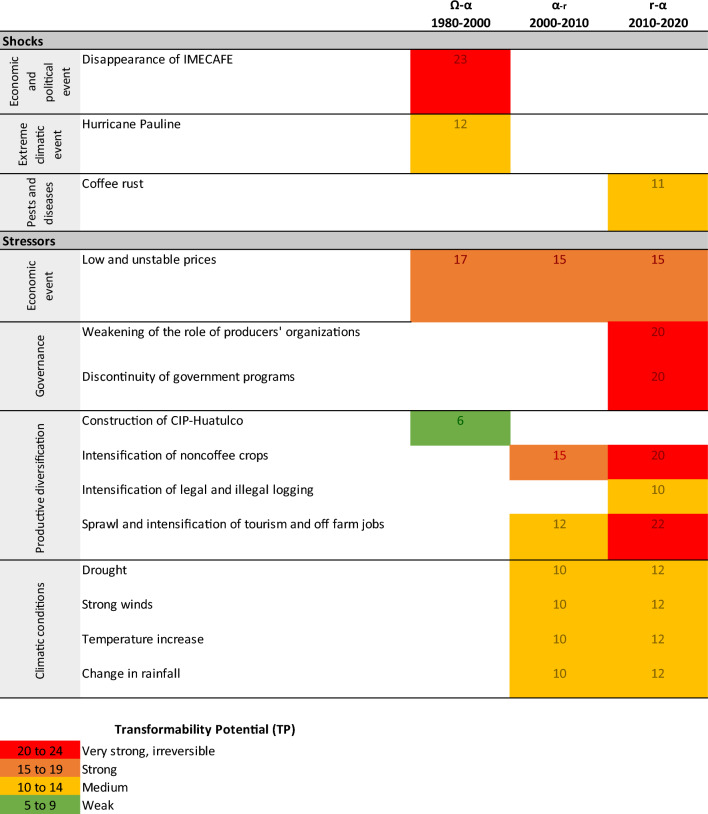
Transformability potential (TP) of stressors and shocks based on the Adaptive Cycle (AC) Own elaboration

### From crisis to rearrangement (Ω-α phase) between 1980 and 2000

The first phase (Ω-α) was a period of creative destruction triggered by the disappearance of the Mexican Institute of Coffee (IMECAFE) (TP equal to 23), which was a shock that paralyzed all components of the AFSES (production, processing, commercialization and environmental and ecological conditions, Fig. 2) throughout the entire watershed and generated the irreversible transformation of the system. In addition, the duration of its effects on economic, political, and organizational issues was incremental and has accumulated to this day. Another shock, Hurricane Pauline (TP equal to 12), struck the entire basin, and its effects were irreversible for coffee production and are still being felt specifically for production and environmental conditions. The main stressors were low and unstable prices (TP equal to 17) and the construction of the touristic center of Huatulco (CIP-Huatulco) (TP equal to 6). Low prices directly affected production, commercialization and environmental conditions throughout the watershed, and their effects were incremental. The construction of CIP-Huatulco in the coastal limits of the CHW initiated a series of gradual changes in production, environmental and ecological conditions; consequently, its effects were incremental.

These driving forces generated a stage of chaos that led to strategies that allowed the AFSES to continue evolving, which mainly were the offer of new jobs in the tourism sector (coping strategy) (which made it possible to supplement the income of the families of coffee growers), the collective organization of coffee farmers (coping strategy), the sales of other crops that grew in the CHW (coping strategy), incursion in new certified coffee markets (adaptive strategy) and the implementation of reconstruction programs to face the damages of the hurricane (coping strategy), among others. On the other hand, according to the testimonies collected (Appendix S2), the residues from washing coffee beans, which generated contamination in streams and rivers during the most productive years, reduced due to the decrease in production, which in the long term improved the supply of drinking water for human settlements. Table 2 summarizes the relationships between the driving forces, their causes and effects, and the strategies adopted to address them in this period.

**Table 2:** Relationships between the driving forces, causes, effects, and strategies adopted in the phase Ω-α, crisis and rearrangement (1980–2000)

Ω-α crisis and rearrangement (1980–2000)					
Driving force (type)	Year	Cause(s)	Effect(s)	Adaptive Strategy/ies (type)	Evidence (testimony)
Construction of CIP-Huatulco (stressor)	1984	This Construction was planned by the federal government to promote mass sun and beach tourism in the region	It encouraged the construction of urban centers and introduced off-farm jobs which impacted production and environmental conditions at the lower part	Alternative employment in jobs related to tourism (Coping strategy)	Testimony no. 2, Appendix S2 Testimony no. 3, Appendix S2 (Jaffee, 2019)
Disappearance of IMECAFE (shock)	1989	Interruption of international coffee agreements. Consequently, national public spending in the agricultural sector was reduced	Political and commercial restructuring to deregulate the production, processing and commercialization of coffee carried out by IMECAFE from 1968 to 1989	Integration of collective organizations of coffee farmers to absorb the functions of IMECAFE in large organizations (e.g. the State Coordinator of Coffee Producers of Oaxaca, CEPCO) (Coping strategy) These organizations came to manage governmental programs. (Adaptive strategy)	Testimony no. 1, Appendix S2 (Paré, 2001)
Low and unstable prices (stressor)	1980s	The international price of coffee suffered several ups and downs	Increase of production costs. Speculation with local coffee prices by local intermediaries. Impoverishment and social exclusion among coffee farmers. First wave of abandonment of coffee cultivation	Sales of noncoffee crops that were already grown in coffee plantations, such as bananas. (Coping strategy) Incursion in international certification seals to obtain price premium (e.g. Fair Trade). (Adaptive strategy) Incursion in the processing of coffee and developed of new brands. (Adaptive strategy)	Testimony no. 4, Appendix S2 (Jaffee, 2019)
Hurricane Pauline (shock)	1997	This hurricane hit the CHW in October 8th under category 4 according to Saffir-Simpson scale	It generated erosion, and loss of native vegetation, springs disappeared, and the soils became more acidic. The productivity and resistance to pests and diseases decreased. Impoverishment of coffee farmers and triggered a second wave of abandonment of coffee cultivation	The government implemented reconstruction programs for the coffee sector that included the distribution of high-yield technological packages. (Coping strategy) Migration was reinforced along the CHW (Coping strategy)	Testimony no. 6, Appendix S2 Testimony no. 7, Appendix S2 (CONABIO, 2020 Vera Cortés, 2005)

### Reconfiguration and innovation (α-r between 2000 and 2010)

In the second phase**,** reconfiguration and innovation (α-r), diverse stressors with incremental and cumulative effects were identified and were characterized by the strengthening of alternative economic activities. These activities were strategies adopted in the previous period and were intensified, generating new conditions that caused secondary effects. The constant presence of low and unstable prices (TP equal to 15) was maintained, resulting from the environment generated by the international coffee markets that impacted local production, commercialization and modified environmental conditions in the entire watershed. The noncoffee crops were a mainstay for the continuity of the AFSES, but they led to clearing the land to cultivate and use agrochemicals across the entire watershed. Thus, the intensification of noncoffee crops (TP equal to 15) impacted the production, commercialization and environmental conditions.

As a result of the construction of the CIP-Huatulco, tourism spread mainly in the lower and middle parts of the watershed due to the increase in visitors, but without planning or regulation, which implied greater demand for water, greater generation of waste and a high disturbance of local ecosystems. This intensification of tourism and off-farm jobs has been perceived as a stressor (TP equal to 12) and has impacted production, commercialization, and environmental conditions. However, the proximity of the tourist center favored the opening of points of sale for locally-produced products. The stressors associated with the change in climatic conditions (TP equal to 10) and their effects mainly affected the production and environmental conditions of the entire watershed.

Local studies, carried out by local government and universities, found that the shade coffee in the CHW contributed to preserve infiltration of water, refuge to biodiversity, and ecological connectivity (Ramos Olivera 2015). Specifically, the highest rates of deforestation were observed outside the altitudinal range of coffee (400 to 1600 m) (SAGARPA and SEDAPA, 2015), where traditional agriculture expanded by approximately 41,000 hectares over the last ten years (Olivera Ramos et al. 2015).

Among the main adaptation strategies in this phase, the noncoffee crops (coping strategy), the incursion into new markets (adaptive strategy), and the coffee processing (adaptive strategy) were maintained from the previous stage. The incursion of coffee growers into ecotourism projects also arose at this stage (adaptive strategy). In addition, government programs and NGO initiatives promoted actions to support shade coffee (such as the payment for the hydrological environmental services program and the initiative water management in watersheds, implemented by WWF Mexico) to encourage the contributions of shade coffee to the conservation of the CHW (adaptive strategy). Table 3 summarizes the relationships between the driving forces, their causes and effects, and the strategies adopted to address them in this stage.

**Table 3:** Relationships between the driving forces, causes, effects, and strategies adopted in phase α–r, reconfiguration and innovation (2000–2010)

α–r reconfiguration and innovation (2000–2010)					
Driving force (type)	Year	Cause(s)	Effect(s)	Adaptive Strategy/ies (type)	Reference (evidence)
Low and unstable prices (stressor)	2000s	The international price of coffee continued to suffer several ups and downs	The social backwardness among coffee farmers continued and was aggravated	Sales of noncoffee crops that were already grown in coffee plantations including oranges, soursop, or cocoa. (Coping strategy) Incursion in international certification markets. (Adaptive strategy) Development of new brands and incursion in the processing of coffee. (Adaptive strategy) Sale of coffee to intermediaries/middlemen. (Coping strategy) Incursion of coffee farmers in ecotourism offers. (Adaptive strategy)	Testimony no.9, Appendix S2 Testimony no. 10, Appendix S2 Testimony no. 11, Appendix S2
Intensification of noncoffee crops (stressor)	2000s	Intensification of nontraditional crops in the shade-grown coffee agroforestry system (e.g., avocado, peach) of commercial importance	Unplanned expansion through the clearing of land and use of agrochemicals	Increasing of use of agrochemicals. (Coping strategy)	Ramos Olivera (2015), SAGARPA and SEDAPA (2015)
Sprawl and intensification of tourism and off-farm jobs (stressor)	2005	Expansion of tourism and off-farm jobs from the watershed’s lower part to the middle part	Urban sprawl in the lower part near the CIP-Huatulco where housing developments have been built for local inhabitants and migrants The urban sprawl increased the demand for freshwater and food, including waste and sewage emissions management. Emigration out of the CHW of young people and the transformation of the heads of household toward single mothers or elderly people	Implementation of governmental programs and nongovernmental strategies that favor the cultivation of shade-grown coffee to maintain water infiltration, refuge for biodiversity, and ecological connectivity. (Adaptive strategy)	Testimony no. 8, Appendix S2 Testimony no. 12, Appendix S2 Ramos Olivera (2015), Lozano-Trejo et al. (2020), SAGARPA and SEDAPA (2015)
Climatic conditions (drought, strong winds, temperature increase, change in rainfall) (stressors)	2000s	Change in local climatic conditions	Decreasing the productivity of coffee plants and increasing disease outbreaks	Unidentified	(Ramos Olivera (2015), SAGARPA and SEDAPA (2015)

### Reorganization to new arrangements (*r*–*α* phase between 2010 and 2020)

In the third phase, reorganization to new arrangements (*r*–*α*) was distinguished by a brief period of recovery; however, various stressors whose effects were incremental and accumulated from the previous stages exerted greater pressure than in previous decades. In addition, the coffee rust plague *(Hemileia vastratix)* hit the AFSES, resulting in a new crisis. First, the presence of low prices was constant (TP equal to 15), affecting the entire watershed and production, commercialization, and environmental conditions, and their effects prevailed incrementally. The role of producers’ organizations lost legitimacy due to allegations of lack of transparency causing members to stop participating in them. This decreased the bargaining power of these organizations in the market and with the government. The weakening of producers’ organizations became a stressor (TP equal to 20) and impacted all the components of the AFSES, with incremental effects.

The discontinuity of government programs became a stressor (TP equal to 20) because the budget allocated for conservation and sustainability in the coffee sector (already weakened) was reduced by the change in government. These programs promoted specific actions such as the reforestation, renovation of coffee varieties, and cultivation of crops associated with agroforestry systems to encourage the infiltration of water provided by coffee plantations and the conservation of native vegetation. Consequently, the absence of these programs affected all the components of the AFSES, and their effects were incremental. The intensification of noncoffee crops (TP equals 20) and the sprawl and intensification of tourism and off-farm jobs (TP equals 22) increased their pressure due to cumulative effects, and both stressors affected the entire basin and all its components.

In this phase, legal and illegal logging emerged as stressors (TP equal to 10) in the upper part of the watershed, and its effects were incremental and affected the components of production, commercialization, and environmental conditions. The stressors related to climatic conditions (TP equal to 12) also increased, and the pressure over the entire watershed increased over the production and environmental conditions. The variability of climate was associated with the appearance of pests and diseases in crops; specifically, the rising temperature triggered the sprawl of coffee rust that shocked the AFSES in 2015 (Avelino et al. 2015); it spread massively and provoked diminished productivity. Coffee rust affected the production and environmental conditions of the AFSES in the entire basin, which suffered irreversible and immediate damage resulting in partially paralyzed activities on the plots. The most affected plots were in the lower zone, and collaboration networks between farmers allowed them to share information to contain the pest; in addition, they shared stocks and capacities to continue marketing and processing coffee. Coffee production has gradually recovered since 2017 because of the knowledge acquired to improve the management of coffee plantations. Coffee rust was considered a shock, and its TP was 11.

Among the main adaptation strategies, coffee farmers increased the diversification of their economic activities through incursion into specialized coffee markets (adaptive strategy), direct sales to national consumers (coping strategy), and offering ecotourism in coffee farms (adaptive strategy). New forms of collective associations among coffee farmers emerged, including horizontal collaboration networks among producers and nonstate actors (NGOs, companies) (coping strategies). Regarding coffee rust, fumigation (coping strategy) and renovation with rust-resistant coffee plants were the main strategies (adaptive strategy). However, coffee plantations required more intensive management, resulting in an increase in the costs of production; NGOs and coffee farmers have been concerned about the possible effects of this change. In addition, although the study period reached 2020, this study did not cover the effects of the pandemic that began in that year, since in the last interview occurred before the effects of COVID-19 were perceived. Table 4 summarizes the relationships between the driving forces, their causes and effects, and the strategies adopted to address them in this decade.

**Table 4:** Relationships between the driving forces, causes, effects, and strategies adopted in phase r-α, from reorganization to new arrangements (2010–2020)

r-α from reorganization to new arrangements, 2010–2020					
Driving force (type)	Year	Cause(s)	Effect(s)	Adaptive Strategy/ies (type)	References (evidence)
Low and unstable prices (stressor)	2010s	The international price of coffee continued to suffer several ups and downs	The coffee cultivation was reduced to 30% lower than in 2010 Imbalance between price and production costs	Intensification of noncoffee crops. (Adaptive strategy) Incursion in new specialized coffee markets. (Adaptive strategy) Development of new brands and processing of coffee. (Adaptive strategy) Sale of coffee to intermediaries and direct sales to national consumers. (Coping strategy) Offer of ecotourism in coffee farms. (Adaptive strategy)	SIAP (2021) Jaffee (2019)
Weakening of the role of producers' organizations (stressor)	2010’s	Lost of legitimacy due to allegations of corruption and lack of transparency Complexity of internal administrative processes	Disappearance of several cooperatives Decrease in the participation of coffee growers	Formation of new collective groups (civil associations, social and private companies, cooperatives) (Coping strategy)	Testimony no. 16, Appendix S2
Discontinuity of government programs (stressor)	2018	Reduction of budget allocated for conservation and enhancing sustainability in the agricultural sector. agroforestry systems	Encouragement of change of crops and land use	Formation of networks of collaboration between farmers, NGOs, enterprises to share knowledge, experience and capacities. (Coping strategy)	Testimony no. 18, Appendix S2 Testimony no. 19, Appendix S2
Intensification of noncoffee crops (stressor)	2010s	As a result of the intensification initiated in the previous decades	Contamination and erosion because of the use of agrochemicals and the land clearing to cultivate	Increasing of use of agrochemicals. (Coping strategy)	Ramos Olivera (2015), SAGARPA and SEDAPA (2015)
Intensification of legal and illegal logging	2010	Bark beetle plague and organized crime	Increase of forestry permits to cut down infested pine trees	Undefined	Testimony no. 17, Appendix S2
Sprawl and intensification of tourism and off farm jobs (stressor)	2010s	As a result of the intensification initiated in the previous decades	Increase of the water consumption, waste generation, and waste sewage emissions. Clearing of land for the construction of spaces required by urban planning	Implementation of nongovernmental initiatives to favor the cultivation of shade-grown coffee to maintain water infiltration, a refuge for biodiversity, and ecological connectivity. (Adaptive strategy)	Testimony no. 17, Appendix S2
Climatic conditions (drought, strong winds, temperature increase, change in rainfall) (stressors)	2015	Change in local climatic conditions	The rising temperature triggered the sprawl of coffee rust	Undefined	Ramos Olivera (2015), SAGARPA and SEDAPA (2015)
Coffee rust plague (shock)	2015	The increase of temperature and low management in the plots (little pruning of shade trees, aged coffee plantations, little soil nutrition)	Devastation of plantations and temporary stoppage of coffee production	Fumigation to control it. (Coping strategy) Renovation with rust-resistant coffee plants. (Adaptive strategy) Collaboration networks between farmers to share information and useful actions to contain the pest. (Coping/adaptive strategies) Intensification of management (pruning and clearing of shade trees, fertilization of soil) (adaptive strategies)	Testimony no. 13, Appendix S2 Testimony no. 14, Appendix S2 Testimony no. 15, Appendix S2

The combination of the accumulation of effects generated over the years and the strategies to absorb the disturbances generated by the driving forces has shaped an interrupted AC (Fig. 4). The adaptive and coping strategies have helped to maintain the fundamental relationships at the core of the AFSES, which is why it has remained in the same state. However, some strategies are currently generating uncertain feedback and trade-offs.

## Discussion

### Proposed framework and resilience

The AC was a useful conceptual tool for understanding the long-term dynamics of change because it described endogenous dynamics resulting in the internal processes of self-organization over time (Sundstrom and Allen 2019); however, the system did not follow the cycle steps. Although the AC supposes that the evolution of an SES can be described as a pattern, it is not the exclusive way in which a system can evolve. In AFSES, the decisions and actions taken by social actors can have multiple effects and trade-offs and are linked to various social and economic processes (Meuwissen et al. 2019). Human agency contributes to the complexity and unpredictability of change processes and the subsequent outcomes (Sinclair et al. 2017); consequently, AFSES can reorganize in multiple pathways. In our study, the CHW showed an incomplete cycle due to the differentiated TP of each stressor and shock as well as the adaptive strategies. We argue that the proposed method is useful for identifying this rupture within the AC, but it is insufficient to explain the implications of having an incomplete cycle.

The permanence of AFSES during the Ω-α phase (1980–2000) and α–r phase (2000–2010) can be explained because the components of the system interact to create conservative structures in time and space, such as the direct incursion of the producers in the processing and commercialization as well as the use of the tourist space of the CIP-Huatulco and other commercial points near the CHW. Another example is the role that government programs and other nongovernmental initiatives played in conserving shade-grown coffee. These structures resulted in persistent interactions that preserve the core of the system as a coffee AFSES (Burkhard et al. 2011). However, in the last stage, r-α (2010–2020), the AFSES confronted more diverse stressors that made it difficult to reach a system responsive and capable of adapting to both internal and external changes (loop formed by r-K). As a result, we have an AFSES that remains unstable and uncertain in the same state without entering another state.

Consequently, it can be considered that the permanence and consequent resilience of the AFSES is based on the cultivation, processing and commercialization of shade coffee and the interactions of the system that have contributed significantly to its maintenance. In this study, adaptive strategies have helped to prevent the system from being transformed. However, the transition pattern between phases (chaos-reorganization-chaos) shows us that the interactions of the system are generating trade-offs and feedbacks with both negative and positive effects that have been exacerbated by the emergence of new driving forces and the associated effects. The accumulation of these effects may lead us to a threshold of transformation of the AFSES, or a new reorganization phase could arise in the coming years.

In resilient agricultural systems, changes have the potential to create opportunities for innovation and new pathways of development (Salvia and Quaranta 2015). For example, regarding the variability of the local climate, studies regarding the capacity of tropical agroforestry systems to address climate change (Simelton et al. 2015; Altieri and Nicholls 2017) have found that on-farm biodiversity enhances a shorter recovery time in the face of climatic disasters. Agroforestry systems can also contribute to the more efficient use of water and improve soil productivity and nutrient cycling (Lasco et al. 2014).

### The role of adaptive and coping strategies

Our proposal was able to observe the importance of adaptive and absorptive capacities for resilience in an AFSES and the strategies that support it. Specifically, these strategies have served to buffer the effects of drivers of change, thus allowing the system to transition through several phases of the same AC without reaching a new system. The literature that has studied the adaptation of coffee AFSES has identified similar strategies, for example, the diversification of crops and economic activities in coffee-producing regions and entry into certified or specialized coffee markets (Eakin et al. 2011; Castellanos et al. 2013). Additionally, community organizations should be strengthened to develop marketing schemes (Fedele et al. 2020) and emigration has been an alternative to confront climatic hazards (Schroth et al. 2009). All these strategies are similar to those taken in the CHW, where a large extent of them have been carried out internally by producers and do not come from external support.

However, our findings showed that those strategies are generating trade-offs that in some cases encouraged new stressful conditions within the AFSES. For example, the intensification of noncoffee crops and tourism and off-farm jobs are favouring land use change, contamination and ecosystem overload. Similar results were reported within existing literature that has studied the historic evolution of AFSES; for example, in monoculture crops, the use of fertilizer and pesticides has increased, and the breakout of pests resulted in an alteration of the ecological conditions (Eakin et al. 2006; Antoni et al. 2019; Babin 2019). In addition, the ongoing processes observed during the last phase are altering the ecosystem and social conditions, enhancing the cross-feedback among its components and scales, such as the response to confront coffee rust and the uncertainty caused by the long-term effects related to the change in management.

Therefore, although it is possible to affirm that the AFSES has been resilient, important doubts arise regarding whether the current conditions are desirable and what are the risks to the future trajectory. For example, the coffee sector has created conditions of poverty and marginalization for local agricultural laborers without land (Higuera Ciapara and Rivera Ramírez 2018). Thus, it is important to understand the effects of adaptive and coping strategies and the limits of the adaptive and absorptive capacities to identify possible trajectories of the system and thus anticipate changes toward a more desirable state. This is especially relevant for digging deeper into trade-offs and the winners and losers that result from these interactions.

### Implications for the coffee agri-food system

The AFSES confronts a broad range of environmental, economic, social, and institutional stressors and shocks (Cabel and Oelofse 2012; Salvia and Quaranta 2015). As we observed in the CHW, the causes and effects of these driving forces are extremely complex due to the spatial and temporal scales involved; additionally, these driving forces have been exacerbated by the characteristics of the regional or local context. Important examples are the intensification of noncoffee crops and sprawl and the intensification of tourism and off-farm jobs. Regarding coffee rust, this plague could be the result of the effects of climate change, such as temperature variability (Avelino et al. 2015; Torres Castillo et al. 2020)**.**

Increased pressure on productive systems is predicted to increase the frequency at which systems cross thresholds and abruptly shift to new states (Yletyinen et al. 2019). In this research, there are combinations of different types of elements, such as characteristics (i.e., biophysical conditions of watershed), driving forces (i.e., hurricanes), and strategies for maintaining livelihood (i.e., tourism and off-farm jobs), that generate trade-offs and selective pressures every day (Santos Prado et al. 2015). These uncertain conditions can push the AFSES toward tipping points. Although crossing thresholds and facing tipping points is inevitable, diversity has played an important role in absorbing disturbances (Meuwissen et al. 2019).

We could observe different examples of diversity in terms of livelihoods, biophysical conditions, and market access that have encouraged the permanence of the AFSES. The coffee AFSES could play an important role in the conservation and identity of its inhabitants. Consequently, to decrease the risk of undesired tipping points or, where necessary, to facilitate transitions across tipping points to a new preferred state, it is necessary to identify the windows of opportunity that triggered planning at different scales. For example, in CHW, there are opportunities to promote sustainable economic diversification that encourages other agroforestry systems or strengthen ecotourism in a planned and orderly manner to provide stable livelihoods for coffee growers and their families. It is also necessary to strengthen collaboration and dialog schemes between farmers and other nonstate actors, including governments, since these strategies have been useful during the crisis.

## Conclusions

Through the theoretical-methodological proposal developed in this study to analyze the historical trajectory of a coffee AFSES, we found that this type of SES do not always comply with a succession process such as the one shown by the AC. The identity of the studied AFSES is constituted by its components and interactions among themselves, which are the production, processing, and commercialization of coffee, and the ecological and environmental conditions on which its production depends. However, over 40 years these components have changed reconfiguring their interactions and although these remain, their conditions are different, and the role of coffee has become secondary to other human activities in the study area. Among the factors that influence this situation are the diversity of stressors that affect AFSES, such as climatic conditions, market uncertainty, changes in public policies, and trade-offs of the strategies adopted to face the driving forces, which have differentiated impacts in space and time. Consequently, the coffee AFSES is in phases of constant reorganization and innovation.

On the other hand, although we identified thresholds and transitions using the AC, resilience thinking needs to provide more guidance on identifying when and where key social variables may reach thresholds that provide a “window of opportunity” or a trigger to drive system change. Additionally, we recognized that the effects generated by adaptation and coping strategies can be negative, and it is important to think about setting limits to adaptation actions. In this sense, the AC is insufficient to analyze the chances of facing several or different alternative trajectories. In addition, it is important to deepen the analysis of the trade-offs and the persistence and transformative capacities of the system to enhance resilience, which was not studied deeply in this research, and it is not clear if the AC is robust and sufficient to carry out this analysis and how the persistence capacity evolve to the adaptive capacity and its challenges.

## Supplementary Information

Below is the link to the electronic supplementary material.

Supplementary file1 (PDF 731 kb)
