# Supplementary material for: The historical trajectory of a coffee agri-food system: A case study in Oaxaca, Mexico
Source: Ambio. 2023 Jul 25;53(12):1847–63. doi: 10.1007/s13280-023-01893-6 (PMC11568096; doi:10.1007/s13280-023-01893-6)
Supplement: Supplementary file 1 — Supplementary file1 (PDF 731 kb) [file 13280_2023_1893_MOESM1_ESM.pdf]

Ambio

Supplementary information

Title: The historical trajectory of a coffee agri-food system: a case study in Oaxaca, Mexico

Appendix S1. Semi-structured interview, guiding questions

QUESTIONS FOR PARTICULAR COFFEE FARMERS

Section 1. First, I would like you to tell me a little about your community.

1. Do you hold a position in your community (or have you held)? What functions did or did it perform?

2. What do you do here in your community (what do you do for a living)?

3. How are they organized to make decisions? and on what issues?

4. Are there environmental problems in your community? Which are? What caused them?

5. What do you currently do? Have you always done the same activity?

QUESTIONS FOR REPRESENTATIVES OF ORGANIZATIONS

Section 1. First, I would like you to tell me about your organization and your work in it.

1. Could you tell me about the creation, objectives and actions of your organization?

2. In which municipalities/localities of the Copalita-Huatulco watersheds do your organization work? How did you choose that site (or sites)? How do they approach the town to start their work? Since when have you been working in those locations?

3. With which organizations are your institution associated (or has been associated) to work in Copalita-Huatulco watersheds?

4. What is your role in the organization? Since when have you belonged to that organization?

5. What activities does your organization carry out (or has carried out) in the Basin specifically regarding coffee growing? In which municipalities/localities? Since when?

QUESTIONS FOR ALL TYPE OF PARTICIPANTS

Section 2. The following questions refer to the coffee activity in the Copalita-Huatulco region.

5. What events have affected coffee in the Copalita-Huatulco watersheds?

6. When did that event happen? In what year? Did it happen again?

9. What were the causes of that events? what were the consequences of that events?

10. What did people do to confront them?

11. Who was involved (or has been involved) in its resolution? Did your institution participate?

12. What do you think are the main challenges for coffee growing in the watersheds?

13. Could you recommend other people like you who can talk to me about coffee growing and the problems it faces in the Copalita-Huatulco watersheds?

Appendix S2. Testimonies of interviewees

Phase Ω - α, 1980 – 2000

1. *"[…] existed IMECAFE, which was the institution that regulated the policy regarding coffee from the financial support for the establishment of crops, drying patios, pulpers, a thousand things that were done with coffee and then assuming the responsibility for marketing […] it disappeared… and most of its functions were passed on to coffee producer organizations […]" (Person 1)*
2. *"When it arrived when we were in the midst of an existential crisis of survival, the Huatulco tourist complex was created and then they began to see that it was better to sell little and little and little of things that were produced there, from a little flower to an herb, whatever, and they were creating very interesting internal markets" (Person 2)*
3. *"[...] tourism created many sources of employment[...]" (Person 2)*
4. *"The first thing they began to sell was... let's say the axis, the pillar that kept coffee in its crisis was the cultivation of bananas..." (Person 2)*
5. *"[…] the coffee business in its most productive years in the 1980s, produced so much, the risk was of contamination of rivers by coffee washing, well, if you log shade to plant coffee plantations but you kept native species. You had your shadow of "ingas" so it was quite controlled, we never had problems of this type and the contamination of the rivers was controllable so you made your waste receiving pits and you could show that the rivers were maintained" (Person 3)*

6. *"[...] we always contaminate the water with "los detapados" (dumping of water used in washing coffee into the river) but since production has fallen, since now there is not as much coffee, it is no longer contaminated as much as 40 years ago" (Person 2)*
7. *"[…] before Paulina there were coffee plants, there were coffee plantations […] of those coffee plantations we should have about 10%. Very little and then the large production units generated large markets, we did not need another activity other than coffee, which created markets and was a large movement […] Years of coffee cultivation were lost, the coffee trees […] left with the Paulina. […] all those 80-year-old plantations are gone, yes? […] that is where the beginning of the coffee end begins." (Person 2)*
8. *"[…] Hurricane Paulina was a tremendous "watershed" […] It even caused many towns that were migrating from the mountains to the coast to pick coffee, they no longer saw that flow of wool and they left elsewhere and never came back. The hurricane upset everywhere: the part of the forests, the salinity of the soil, they say that as a result, the palms died. But it was also an issue that had a very strong social impact […] I had to go out and work in something unrelated to coffee […]. (Person 3)*

Phase α – r, 2000-2010

9. *"For me, the Huatulco project is a formidable project that came to strengthen us, but it does require a lot of water" (Person 2)*
10. *[…] organic coffee certifications, […] the markets, the qualities, all of that has had to be adapted and whether to advance in the value chain […] possibly at the beginning, as there was more volume of coffee, we were more focused to an export issue and in export we were rewarded with certification seals [...] we entered into a dynamic in 2004 to 2007 of certifying all our producers [...]. (Person 3)*
11. *[...] yes, I have the wet mill, I have the dry mill, I have the toaster [.] and I have the machines to prepare the coffee, [...] we had to get into that added value (Person 4)*
12. *"[…] you don't see the big coffee trucks anymore. But if you see one in those squares […] which are Mondays in Pochutla […] you see 10, 20 people who are going to sell their ground coffee packaged in their plastic bags […] there are those who can carry 2 kilos, but there are those who can carry 100 kilos (Person 2)*
13. *"[…] In 2009, perhaps from 2009 to 2012, [...] there we tried to make the organization independent, we began to manage projects as a system, not so much as an organization, but rather the product system and its renewal project… the product system and its technical assistance project. And they became interesting amounts, because the people from the Ministry of Agriculture gave us that possibility, so we became a little more independent, we were already hitting each other here with the people from the state [...]" (Person 3)*

Phase r - α, 2010-2020

14. *“[…] many said, "it was a great teacher (rust), [it taught us] that [we have to] renew ourselves and really be producers [...] not just be collectors" (Person 5)*

15. *“[…] this time the rust comes to us for the second time and in a brutal way we have aged and poorly worked coffee plantations. Then it coincided that the super fungus arrived because it entered Chiapas from all sides and got into all the regions of the state, the high and cold humid area was spared a little, logically and it hit much harder where you had coffee plantations where the sun or the heat, and everything made the fungus develop faster. So right now, there is not a single region that is free, all the regions have rust on a greater or lesser scale in some areas it is completely gone, in others it was a 50, 60% blow and left coffee plantations in bad condition. This was from […] 2014 to 2015 […] and from then on every year there are mirages all of a sudden, you say “look there it didn't hit this section it didn't hit it, oh, we got rid of it!, let's see what variety is, we are going to take care of it” and boom! next year, it turns out that he ended up throwing it out [...]” (Person 3)*

16. *“[…] rust killed us coffee plantations killed us overnight as if you had poured burnt oil on them. There was no more coffee to cut so our process of renewing coffee plantations to reactivate economically takes 4 - 5 years... 4 - 5 years, boy! Well, there is no wallet that can hold […]. Suddenly once again a blur [...] and a new account again from scratch we are going to redo everything and that is how we have been in recent years as if reinventing something new each time and while the deterioration continues aggressively.” (Person 3)*

17. *“[…] companies that […] buy the coffee are interested in knowing not only what variety you are producing but also your production methods, all of this implies something very important for them, in fact right now you may not have an organic certificate, but they are still interested totally what your methods are not only in the primary part but in the processes that can also be contaminated. [...] then with these types of issues, in these types of issues the purchasing companies dictate a little bit the issue of how to carry out your activities in the field in an organized manner [...]” (Person 3)*

18. *"[...] it is detected as a strength of the basin, let's say [...] the good part, is precisely that belt in the part of the middle forest, which is where the coffee plantations of that area are located, and they are in the order of about 25,000 hectares [….] coffee plantations play a very important role in the middle basin, and the change in land use is taking place in the upper basin, so that is what is serious because it is causing a process of desiccation and soil impoverishment, which could alter the support service a good [part]” (Person 6)*

19. *“[…] right now, if they cut back on something, it is the environmental issue, but tremendous, that is, all the projects that were for this matter to generate projects, including productive ones in the basin areas were removed. There are no more, and I insist that if the economic part is not there then the risk is very high. (Person 3)*

20. *"How does the president of the republic expect ecosystems to be conserved or forest fires to be fought when the budget for the environmental sector has been reduced by a large percentage?" (Person 7)*

Other consulted bibliography

Comisión Nacional para el Conocimiento y Uso de la Biodiversidad (CONABIO). 2022. *Hurricane Paulina Case | Mexican Biodiversity,* from https://www.biodiversidad.gob.mx/monitoreo/m_ecosistemas/huracan-paulina (In Spanish, Web material)

Jaffee, D. 2019. *Harvesting Justice: Fair Trade Coffee, Sustainability and Survival*. https://doi.org/10.15760/soc-01 (In Spanish, Book)

Lozano-Trejo, S., Olazo Aquino, J., Pérez-León, M. I., Castañeda-Hidalgo, E., Díaz-Zorrilla, G. O., & Santiago-Martínez, G. M. 2020. Infiltration and runoff of water in soils of a basin in southern Mexico. *Revista Terra Latinoamericana*, *38*, 57–66. https://doi.org/10.28940/terra.v38i1.443 (In Spanish, Journal article)

Olivera Ramos, F., Lozano Trejo, S., Castañeda Hidalgo, E., & Brena García, G. 2015. *Ecological quality, natural fragility and vulnerability (climatic and anthropogenic) of the Copalita River sub-basin.* (In Spanish, Report)

Paré, L. P. 1990. Losing weight for INMECAFE or for small coffee producers?. *Sociológica México*, *13*. (In Spanish, Journal article)

Vera Cortés, G. 2005. Social vulnerability and expressions of disaster in the district of Pochutla, Oaxaca. In *The social construction of risks and Hurricane Paulina,* ed. García Acosta, V., 35-151. México: Ediciones de la Casa Chata. (In Spanish, Book)

Secretaría de Agricultura, Ganadería, Desarrollo Rural, P. y A. and Secretaría de Desarrollo Agropecuario, Forestal, P. y A. (SAGARPA and SEDAPA). 2015. *Integrated socioeconomic diagnosis of the Copalita River sub-basin* (In Spanish, Report)

Appendix S3. Codifying system.

The Table S1, shows the codifying system used to analyze the data. From left to right, the main categories were the roof of the analysis which began to classify the information to 1) identify shocks and stressors, and 2) identify their influence over each component of the SES's subsystems. Then, the subcategories were divided into 3) processes, situations, and actors, 4) adaptive and coping strategies, 5) spatial scale, and 6) temporal scale. First, subcategories 3 and 4 identified the effects of the drivers on the main relations of the SES, its processes, and the adaptive and coping strategies. Second, these subcategories were classified with respect to categories 5 (spatial scale) and 6 (temporal scale). Thereby, the subcategories 3 and 4 were replicated for each stressor and shock (category 1) as well as with respect to each component (category 2) according to each part of the basin (5) and temporal scale (6).

Codifying system

Main categories		Subcategories			
**Driving forces (1)**	**Components of SES (2)**	**Processes, situations & actors (3)**	**Adaptive & coping strategies (4)**	**Spatial scale (5)**	**Temporal scale (6)**
Shocks/Stressors	Environmental & ecological conditions	Pest & diseases			
		Ecosystem services			
		Environmental conditions			
		Cultivation practices	Economic activities ex ante		
	Production	Importance	Economic activities ex post		
		Decrease	Public policies		
		Coffee varieties	Migration	Low part	Short term
		Price & production costs	Diversification	Middle part	Long term
		Financing	Intensification	Upper part	Ongoing
		Coffee farmers organizations	Producers' organizations		
	Processing	Processing	Private initiatives (individual)		
	Marketing	Marketing			
		Public policies			
		Coffee farmers organizations			

Table S1. *Codifying system.*
